# Harnessing TGF-β signaling to improve testicular organoid development from dissociated testicular cells

**DOI:** 10.1186/s13287-025-04513-0

**Published:** 2025-08-20

**Authors:** Seyedeh-Faezeh Moraveji, Saiedeh Erfanian, Mohammad Hossein Ghanian, Hossein Baharvand

**Affiliations:** 1 Department of Tissue Engineering, Faculty of Basic Sciences and Advanced Technologies in Medicine, Royan institute, ACECR, Tehran, Iran; 2 Department of Cell Engineering, Cell Science Research Center, Royan Institute for Stem Cell Biology and Technology, ACECR, Tehran, Iran; 3 Department of Developmental Biology, School of Basic Sciences and Advanced Technologies in Biology, University of Science and Culture, Tehran, Iran; 4 Department of Stem Cells and Developmental Biology, Cell Science Research Center, Royan Institute for Stem Cell Biology and Technology, ACECR, Tehran, Iran

**Keywords:** Testicular organoid, TGF-β signaling, Small molecule, Seminiferous tubules, Steroidogenic activity

## Abstract

**Background:**

Testicular organoids (TOs) have generated great interest in reproductive biology as a reliable experimental tissue model for pharmaco-toxicology studies and therapeutic applications. However, current TOs mostly fail to recapitulate the native testicular architecture and function, likely due to an imbalance between the spermatogonia and functional niche cells. TGF-β signaling pathway is a critical regulator of testis development that can be harnessed to regulate the testicular cells’ behavior. Based on our previous finding on the crucial role of TGF-β inhibition in promoting spermatogonia proliferation and differentiation, we have developed a novel approach to improve TO development from dissociated testicular cells.

**Methods:**

The testicular cells were isolated from prepubertal mice, encapsulated within inner core of Matrigel-based core-shell hydrogel droplets hanging from filter inserts, and exposed to the small molecule TGF-β inhibitors, SB431542 (SB) or LY2157299 (LY).

**Results:**

According to our results, TGF-β inhibition considerably improved formation of spherical-tubular structures (STSs), resembling the compartmentalized architecture of native testicular tissue, as indicated by increased number, size and average area of the STSs. The TGF-β inhibitor-derived TOs (TiTOs) revealed more profoundly a tissue-specific spatial expression pattern of testicular markers and superior steroidogenic activity in response to gonadotropin stimulation (3.69-fold and 3.00-fold vs. the untreated control in the SB and LY-treated groups, respectively). The stimulatory effects of the TGF-β inhibition were attributed to the promoted proliferation of cells, as demonstrated by up-regulation of cell cycle promoting genes, down-regulation of proliferation inhibitor genes, and up-regulation of proliferation genes, as well as increased number of proliferative germ cells in the treated TOs.

**Conclusion:**

This work presents a simple and efficient method for development of well-organized and functional TOs which can be investigated as a complementary treatment with any other TO culture systems.

**Supplementary Information:**

The online version contains supplementary material available at 10.1186/s13287-025-04513-0.

## Introduction

Male infertility accounts for 50% of all infertility cases and affects approximately 9–16% of men worldwide. Our knowledge of male reproductive biology and the regulatory mechanisms of spermatogenesis is incomplete and hinders research into male infertility as well as its diagnosis and treatment. Therefore, there is a significant need for improved technologies to create experimental models for studying male reproduction and developing novel drugs to treat male infertility [1–3]. Current methods for assessing pharmaceutical compounds and environmental gonadotoxicity rely mostly on animal models, which are limited by challenges such as the need for large numbers of animals, high costs, variable drug effects among species, and ethical issues [4]. Alternatively, two-dimensional (2D) cell culture systems are inefficient due to their inability to mimic in vivo testicular microenvironment and cell-cell communications [2, 5].

In recent years, several attempts have been made to recapitulate the testicular microenvironment in vitro using three-dimensional (3D) organ-like structures called testicular organoids (TOs). These advanced 3D culture systems are considered as an intermediate platform between 2D culture systems and animal models and have attracted great interest as a more reliable model in reproductive biology for various applications such as oncology, toxicology, regenerative medicine, and drug discovery [1, 6, 7]. Numerous cell culture systems have been developed to generate TOs by recapitulating the testicular microenvironment using a series of multicellular co-cultures and permissive matrices [8, 9]. These culture systems are intended to provide suitable conditions for cellular reorganization through scaffold-free culture of multicellular aggregates [4, 10] or encapsulation of individual cells in hydrogel scaffolds based on different natural and synthetic biomaterials [9, 11–17]. Despite significant advances, these culture systems mostly lacked structural features characteristic of normal testicular tissue [6, 18, 19], likely due to an imbalance in the population of major testicular cell types and inefficient cell proliferation and differentiation during TO formation [20].

The first important events that regulate the development of testis cords during development are the proliferation and differentiation of germ and Sertoli cells (SCs) [9, 21]. Thus, triggering these critical events during TO development through exogenous signal manipulation could be helpful in overcoming the challenges associated with TO development. Our previous study shed light on the fundamental role of transforming growth factor-beta (TGF-β) inhibition in the expansion of mouse and human spermatogonia. We found that SB431542 (SB), a TGF-β inhibitor (TGF-βi) small molecule, enhanced germ cell proliferation and accelerated spermatogenesis in testicular tissue of infertile mice. The regulatory function of TGF-β signaling pathway in the testis development has been known in spermatogenesis, cellular behaviors of Sertoli, germ and peritubular myoid cells and the secretory function of LCs [22–25]. Based on these evidences, we hypothesized that inhibition of TGF-β signaling could promote the reorganization of dissociated testicular cells into seminiferous tubule-like structures and improve TO development by triggering key cellular processes linked to testis development. To address this hypothesis, the dissociated mouse testicular cells were cultured within the inner core of a well-known Matrigel-based core-shell hydrogel and exposed to TGF-β inhibitors, SB, or its agonist LY2157299 (LY) to reveal whether TGF-β inhibition affects TO formation capacity and functionality.

Through inhibiting TGF-β signaling, we achieved well-organized TOs with spherical-tubular structures (STSs) and improved features regarding cellular reorganization, spatial gene expression pattern, and hormone secretion. Our results further highlighted the important role of TGF-β signaling in testes development, which can be targeted in upcoming efforts for male infertility treatment and improved development of TOs.

## Materials and methods

### Preparation of testicular cells

In order to obtain total testicular cell suspensions, eight testes from 14-day-old mice were digested for each experiment as previously described [26]. Euthanasia of mice was performed using gradual-fill CO₂ inhalation in accordance with institutional animal care guidelines to minimize distress and ensure a humane procedure. Briefly, the mice were placed in a sealed chamber, and CO₂ was introduced gradually at a displacement rate of approximately 30–70% of the chamber volume per minute. All animal care was in accordance with the approval of the Royan Institutional Review Board and Institutional Ethical Committee (license number IR.ACECR.AEC.1401.067).

### Testicular organoid culture and treatment with small molecules

To establish TO culture, a 3D multilayer culture method was used based on previous report with some modifications [6]. The procedure involved embedding dissociated testicular cells in a layer of Matrigel (Sigma-Aldrich, USA), positioned between two layers of the same matrix without cells, on top of a hanging cell insert (Corning, 3450, USA), which formed a three-layer system (core-shell). A total of six Matrigel droplets were placed on each hanging cell insert in a 6 well plate. To optimize the conditions for TO culture, different cell densities (22 and 44 million cells/ml) and cell maturation stages (neonatal and prepubertal cells from mice aged 3–5 and 14 days) were examined. The basic culture medium used for TO culture was α-MEM medium (Hyclone, USA) supplemented with penicillin/streptomycin (0.5%, Invitrogen, USA), l-glutamine (2 mM, Invitrogen, UK), non-essential amino acids (0.1 mM, Invitrogen, UK), beta-mercaptoethanol (0.1 mM, Sigma-Aldrich, USA), KnockOut serum replacement (KSR, 10%, Invitrogen, USA), Retinoic Acid (RA, 100 mM, ICN), melatonin (10^− 7^ M, Sigma-Aldrich, USA), FSH (5 IU/l, CinnaGen, Iran) and hCG (5 IU/l, Health Biotech Ltd, India). The 3D co-cultures were incubated at 35 °C with 5% CO_2_ and half of the medium volume was substituted every other day. After 5 days of culture, the medium was supplemented with the TGF-β inhibitors SB (SB431542,10 µM, Cayman) or LY (LY2157299, 5 µM, Medchem Express) for 11 days. The cultured testicular organoids (control and treated groups) were assessed morphologically using phase-contrast microscopy during the culture period.

### Live/dead assay

At the end of the culture, cell viability within the organoids was determined using acridine orange (AO) and propidium iodide (PI) (AO/PI) staining (Sigma-Aldrich, USA). First, stock solutions of AO (670 µmol/L) and PI (750 µmol/L) were prepared separately in PBS and stored in the dark at 4 °C. Before use, the working solution was prepared by mixing AO (0.01 ml) and PI (1 ml) solutions and diluting 10-fold with PBS. The cell-laden Matrigel samples were washed with PBS, incubated in the AO/PI solution for 10 min, and observed under a fluorescence microscope (Olympus, IX71, Japan). Viable and non-viable cells were stained green (AO) and red (PI), respectively.

### Migration assay

The relative migration area for each culture condition was evaluated according to the previous report [6]. Briefly, the area occupied by cells was measured from day 0 to day 10 using the image analysis program ImageJ software.

### Histology evaluation of testicular organoids

For histological assessment, samples were fixed in 4% paraformaldehyde (Sigma-Aldrich, USA) for 2 h at 4 °C followed by 30 min at room temperature (RT). The samples were pelleted in 2% agar, then dehydrated with alcohol, immersed in xylene, and embedded in paraffin. The paraffin-embedded fragments were cut into 5 μm-thick sections, followed by deparaffinization and rehydration steps: dewaxed in xylene, hydrated in decreasing concentrations of ethanol, and stained with hematoxylin and eosin (H&E, Sigma-Aldrich, USA). For quantitative histological analyses, TOs were evaluated for STS frequency, average STS area, and lumen efficiency per cross-section of organoids. The number and area of STS were measured by ImageJ software in images captured at 100× and 200× magnification. The area of ​​an STS cross section (A_c_) was evaluated using the equation A_c_ = *πD*^2^/4, where *π* was 3.142 and *D* was the mean diameter of the STSs [27]. Quantification of the lumen efficiency was carried out based on a standard procedure [9]. Briefly, the entire area of one STS containing a lumen, and the area of the lumen were determined using ImageJ software. The percentage of lumen efficiency was determined by dividing the area of the lumen by the area of the STS containing the lumen. Histological observations were performed using a conventional bright-field microscope (Olympus, SZX12, Japan). The experiments were repeated in at least three independent biological experiments and the supplementary information for the histological analyses is presented in Supplementary Table S1.

### Immunostaining

Immunostaining for specific markers was performed to evaluate the distribution of testicular cell types (Germ cells, Sertoli, Leydig, and peritubular myoid cells) within TOs, immunostaining for specific markers was performed. For α-SMA/Ddx4, organoids were imaged whole mount, but for other markers organoids were imaged in 5 μm tissue sections. For immunostaining of the sections, samples were deparaffinized in xylene and rehydrated in descending series of ethanol series. The antigen retrieval process was performed by incubating the slides in sodium citrate at pH 6 or TrisEDTA at pH 9 buffer at 95 °C for 15 min. The samples were then permeabilized with 0.1% Triton X-100 for 10 min. The nonspecific reaction was blocked with 10% normal serum and 4% bovine serum albumin for one hour at 37 °C. Samples were incubated with primary antibodies at the desired dilution overnight at 4 °C. The primary antibodies used in this study were anti-α-SMA (1:200, Abcam, ab7817, USA), anti-Ddx4 (1:100, Abcam, ab13840, USA), anti-PCNA (1:100, BD, 610665, Canada), anti-Vimentin (1:200, Sigma-Aldrich, V6630, USA), anti-ZO-1 (1:50, Abcam, ab216880, USA), anti-3β-HSD (1:100, Santa-Cruz, SC-30820, USA), and anti-β-catenin (1:100, Santa-Cruz, SC-59737, USA). After washing the samples with 0.1% Tween-20 in PBS for 5 min, secondary antibody incubation was performed for 1 h at 37 °C. The secondary antibodies used in this study were donkey anti mouse (Abcam, ab150105, USA), goat anti rabbit (Abcam, ab98509, USA), donkey anti rabbit (Invitrogen, A21206, USA), donkey anti goat (Invitrogen, A11057, USA), and goat anti mouse (Abcam, ab98805, USA). The same staining procedure without primary antibodies was applied for the negative control. DAPI (4′,6-diamidino-2-phenylindole) was used to label the nuclei.

For wholemount staining, we used the previously described protocol [6]. The cell-laden Matrigel samples were removed from the transwell inserts using a scalpel. Samples were fixed in 4% paraformaldehyde (Sigma-Aldrich, USA) for 2 h at 4 °C followed by an additional 30 min at RT. After fixation, antigen retrieval was applied in 10 mM aqueous sodium citrate solution and 0.05% Tween 20 pH 6 for 15 min at 95℃. After cooling for 30 min, permeabilization was carried out by washing the samples three times with PBS containing 1% Triton X-100 for 30 min at RT. Blocking was achieved by incubating the samples twice with PBS, 1% Triton, 10% Normal Donkey Serum and 0.2% sodium azide for 1 h at RT. Primary antibodies incubated for 3 days at 4 °C. After washing, secondary antibody incubation was performed for 3 days at 4℃. Counter-staining was performed by adding DAPI to the secondary antibody solution on the second day of incubation, resulting in a final concentration of 4 mg/ml. After whole-mount staining, samples were examined using confocal laser scanning microscopy (CLSM; LSM800, Carl Zeiss, Germany). To create a superficial 3D representation of the TOs, a Z-stack command was utilized. In this process, images were captured at 7 μm intervals from samples with a thickness of approximately 100 to 150 μm.

### Cell number quantification within organoids

To quantify the number of Ddx4^+^/PCNA^+^ cells and β-catenin^+^/3β-HSD^+^ cells, a total of three organoids in each group were stained and analyzed using ImageJ software. The number of immunolabeled cells was manually counted in images taken at 200× magnification with a fluorescence microscope and presented as a percentage relative to the number of DAPI-stained cells. A ratio between the number of cells stained for a marker of interest and the total number of cells (DAPI^+^ cells) was calculated to allow comparison of different groups. The experiments were repeated in at least three independent biological experiments with the supplementary information’s presented in Supplementary Tables S2 and S3.

### Hormonal assay

To assess the steroidogenic activity of TOs, the culture media of organoids were collected before and after gonadotropin exposure to measure testosterone levels (ng/ml^*−* 1^) using an ELISA kit (Biocompare, KET 0001) according to the manufacturer’s instructions. We added hCG (5 IU/L) and FSH (5 IU/L) to the organoid culture media from the ninth day of culture until the end of the experiments. After incubation, cell culture supernatant was collected from both treated and untreated organoids. For this experiment, a total of 18 organoids were used per time point for each group. All samples were analyzed in duplicate. A standard curve was created and used to determine testosterone concentration.

### Quantitative RT-PCR for gene expression analysis

Total RNA was extracted from TOs using the Qiagen RNeasy plus universal mini kit according to the manufacturer’s instructions (Qiagen, Germany). The cDNA was synthesized and amplified using a cDNA synthesis kit (Parstous, Iran) according to the manufacturer’s instructions. Then, 25 ng of the synthesized cDNA was used for quantitative RT-PCR (qRT-PCR) analysis using a SYBR Green PCR Master kit (Amplicon, Denmark). Samples were analyzed for the gene expression of *Gapdh* (housekeeping) and cell-specific markers: *Grfα1*, *Ddx4* and *Oct4* (Germ cell), *Smc1*b and *Prm1* (differentiated cell), *Vimentin*, *Sox9* (Sertoli cell), *Ki67*, *Pcna* (Proliferating genes), and TGF-β signaling target genes. Primers used in this experiment are presented in Supplementary Table S4. The relative gene expressions levels were normalized to those of *Gapdh.*

### Statistical analysis

All experiments were repeated at least in three independent biological replicates and expressed as mean ± SD. In order to determine the statistical significance of our data we used GraphPad 320 Prism software (GraphPad Prism 7, Boston, USA). Statistical analyses were performed using Student’ s *t-*test and one-way ANOVA followed by Dunnett’s or Tukey’s *post-hoc* analysis. P-values < 0.05 were considered significant.

## Results

### Establishment of testicular organoid culture

To develop TO culture, dissociated testicular cells were encapsulated in Matrigel-based hydrogel with two different configurations: Homogeneous and core-shell hydrogel. We observed that cultured cells in the core-shell system showed signs of self-assembly and organization. In contrast, cells in the homogeneous only formed densely packed aggregates (Supplementary Fig. 1A and B). To determine the proper culture conditions, we examined testicular cells at two different densities (22 and 44 million cells/ml) and two different maturation stages (neonatal and prepubertal cells from mice aged 3–5 and 14 days). We selected the condition of 44 million cells/ml isolated from 14-day-old mice due to its greater potential for cellular self-assembly (Supplementary Fig. 2).

### The effect of TGF-β inhibition on the reorganization of testicular cells

**Fig. 1:**
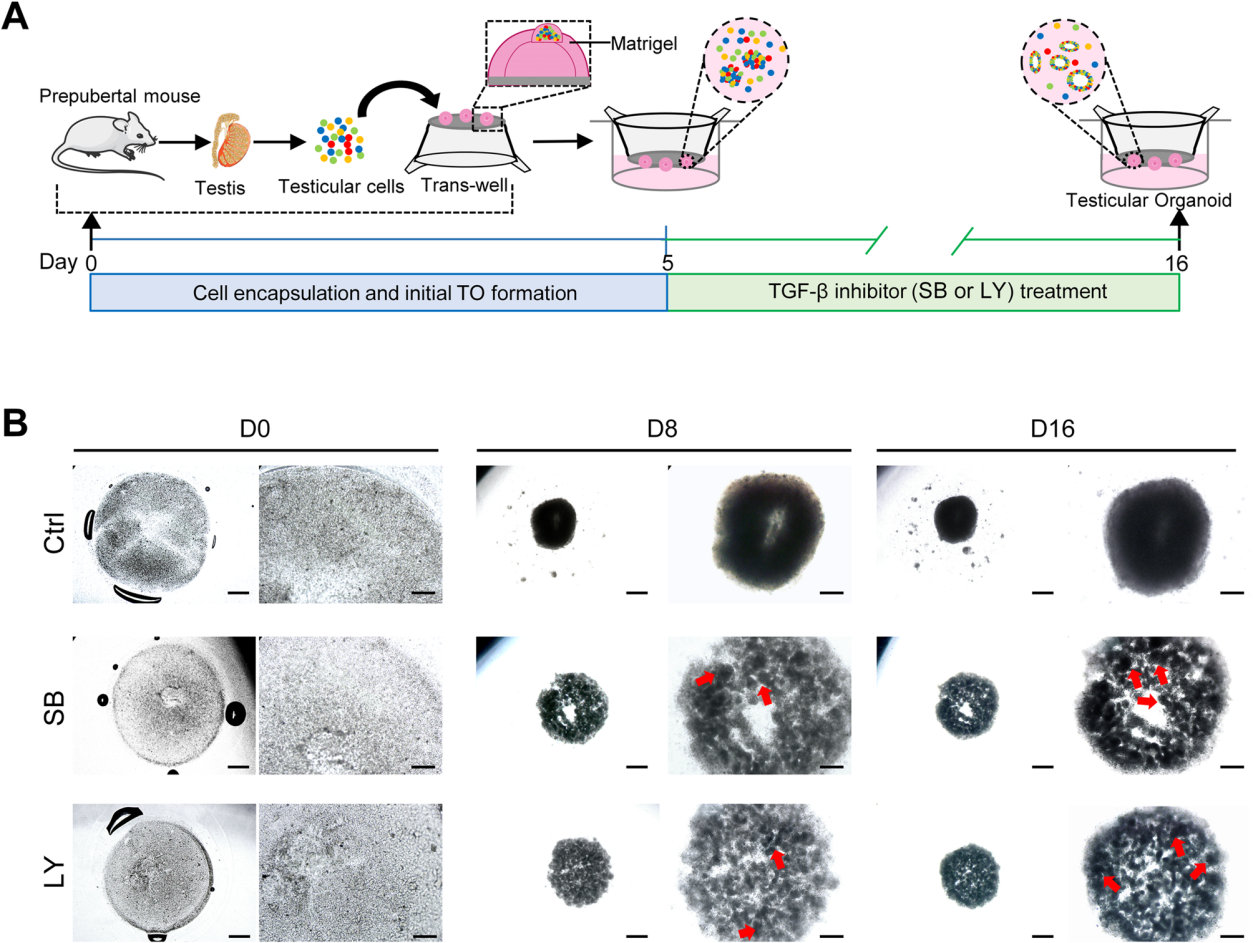
Gross observation of TO development during TGF-β inhibition. (**A**) Schematic representation of TO culture and small molecules (SB or LY) treatment. The small molecules were added to the culture on day 5 and continued to be added on subsequent days until the end of the 16-day culture period. (**B**) Representative bright-field images of TiTOs on days 0, 8, and 16 of culture. The images illustrate cellular reorganization with different patterns during 16 days of culture, both with and without TGFβ inhibitors. Distinct compartments were observed only in the treated groups on day 8 and were more prominent on day 16 (red arrows). In contrast, cells in the control group formed compact and dense aggregates with no apparent compartmentalization. (Right panel: High magnification). TO: testicular organoid; TiTO: TGF-β inhibitor-derived testicular organoid; SB: SB431542; LY: LY2157299. Scale bars: 500 μm, high magnification: 200 μm

A schematic diagram showing the culture method used in this study is illustrated in Fig. 1.A. Testicular cells were cultured within the Matrigel-based core-shell system for 5 days to establish an initial cellular assembly, followed by treatment with SB or LY for an additional 11 days. No significant cell death was observed during the entire 16 days of culture, and viability of the treated culture with TGF-β inhibitors was like that of the control group (Supplementary Fig. 3). Within one day of culture, the cultured cells began to compact and formed aggregates and by the fourth day, the aggregates showed gradual aggregation into tubular structures (data not shown); However, on days 8 and 16, in cultures treated with TGF-β inhibitors, developing distinct tubule-like structures were observed (Fig. 1B). Conversely, the aggregates in the untreated group, exhibited greater compactness during the following culture days. To examine the kinetics of cell organization, the migration profiles of cultured cells were followed for 10 days. Our observations revealed a similar cell migration pattern; However, differences between groups were observed on days 6 and 10 (Supplementary Fig. 4).

### The effect of TGF-β inhibition on the reconstruction of tubular structures

**Fig. 2:**
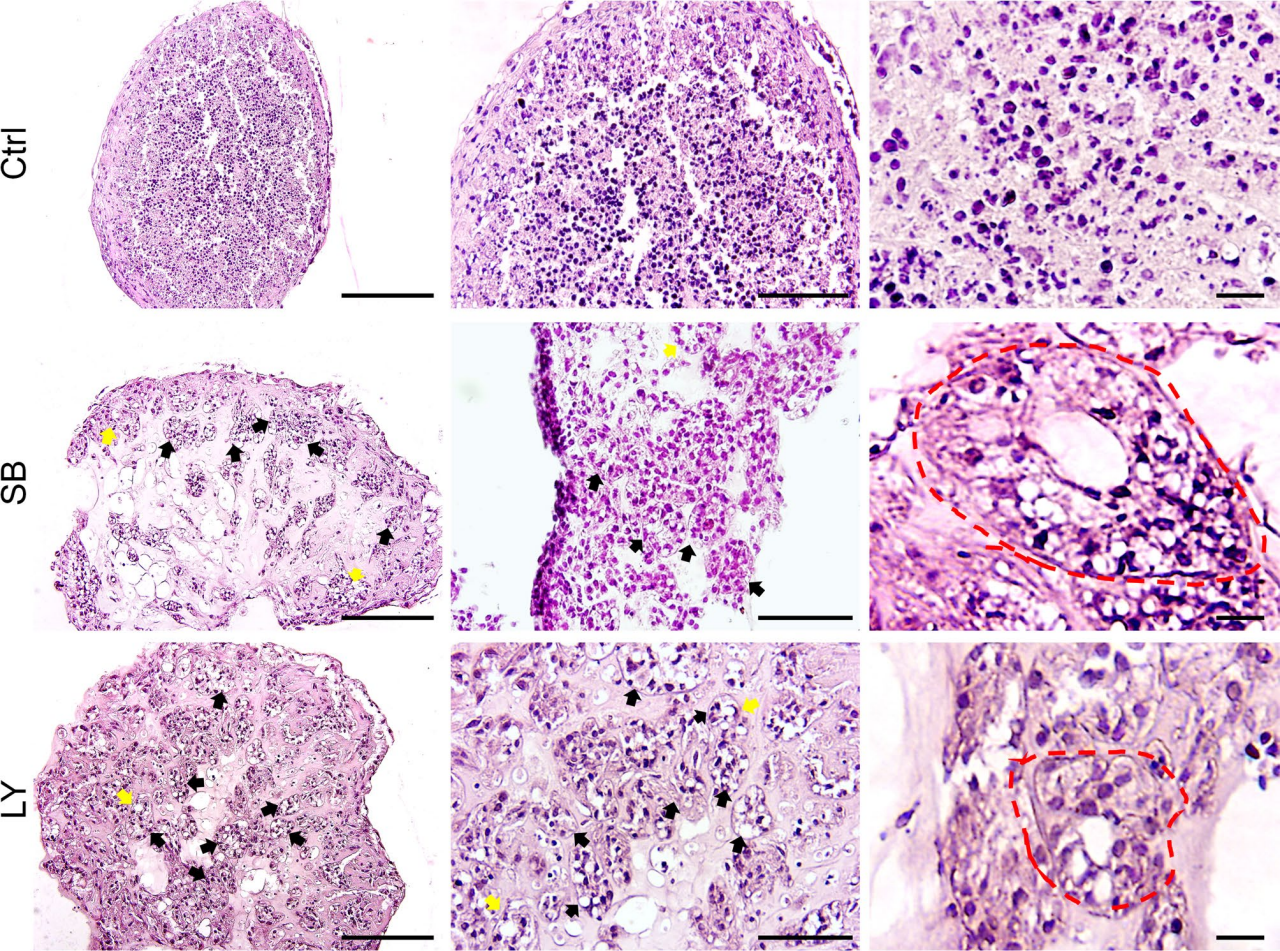
Histological examination of TOs. Paraffin sections of TOs on day 16 were stained by H&E and examined at both low and high magnification. The results showed the reorganization of cells into distinct STSs (represented by black arrows), with some STSs having a lumen-like inner core (represented by yellow arrows) in TiTOs. Inhibition of TGF-β significantly improved the reconstruction of STSs from testicular cells and developed compartmentalized tubule-like structures. In contrast, the control group showed a compact cell assembly without sign of tubule formation (Right panels: High magnification). Scale bars: 200 μm, high magnifications: 100 μm and 20 μm

Histological examination revealed that TGF-β inhibitors enhanced the reconstruction of tubular structures from dissociated testicular cells. As evident in Fig. 2, TGF-β inhibition led to the development of organoids (TiTOs) with well-defined and distinct tubular structures. In TiTOs, the dissociated testicular cells were arranged and organized in discrete STSs, exhibiting a compartmentalized testis-like architecture. These well-defined STSs were found in larger numbers in the TiTOs than in the untreated ones. In transverse histological sections, most of the STS in TiTOs had a round shape, while some other STS, especially in the LY group, had cord-like structures resembling the primary sex cords found in testis development in vivo (Supplementary Fig. 5). Interestingly, we observed clearly defined lumen-like structures in the TiTOs, whereas the control group had either no or very few lumens.

**Fig. 3:**
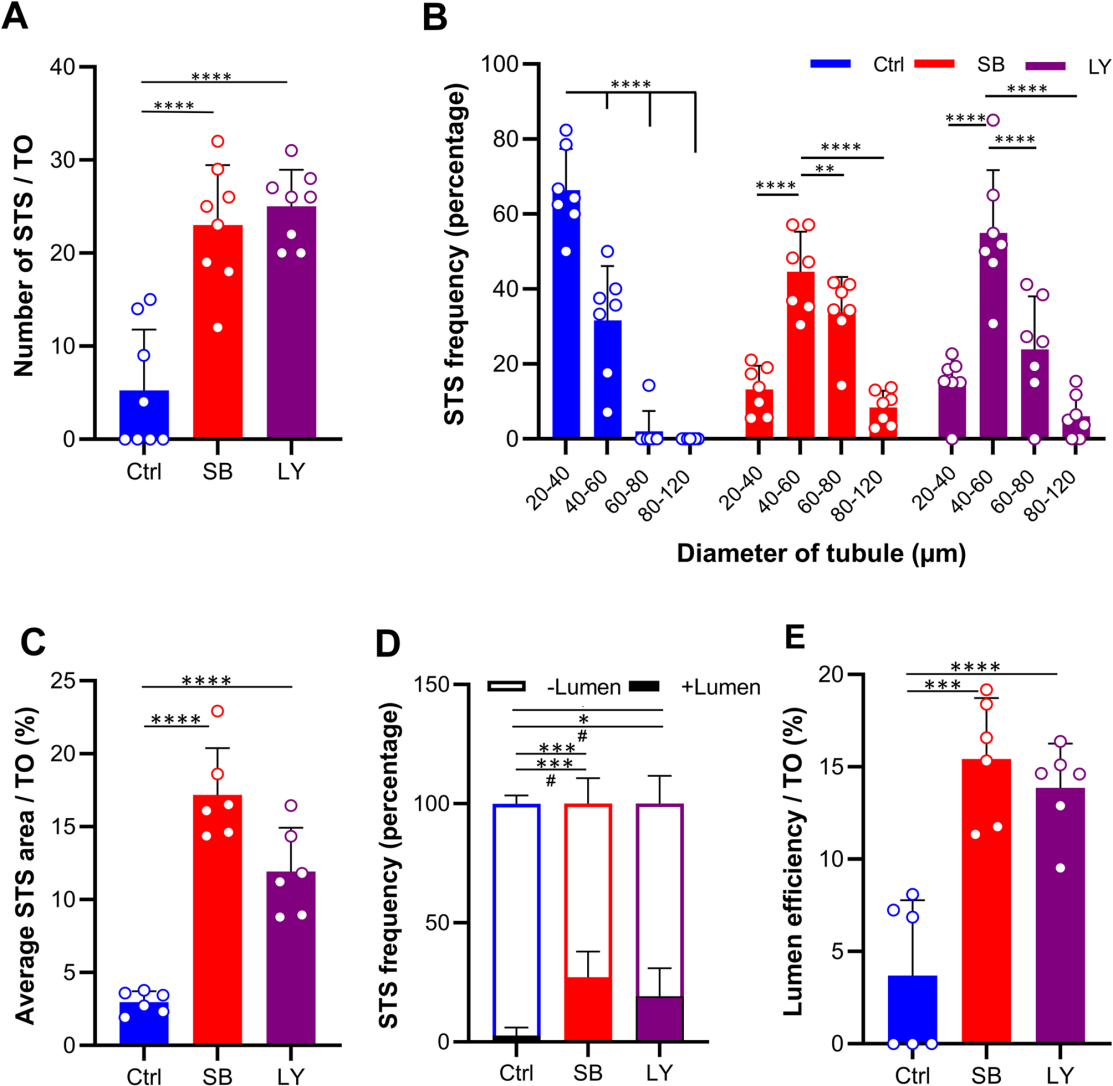
Quantitative morphometric characteristics of TOs based on histological examination. (**A**) TGF-β inhibition resulted in a significant increase in the number of STSs in TiTOs. **(B**) The STSs observed in the TiTOs were larger than those in the control group (the most frequent size ranges of 40–60 μm vs. 20–40 μm, respectively). (**C**) The average STS area was significantly higher for the TiTOs. (**D**) Lumen-bearing STSs were found more profoundly in the TiTOs. (**E**) The TiTOs exhibited a significant increase in lumen efficiency. Data are collected from 6–8 TOs, presented as mean ± SD, and analyzed by one-way ANOVA (Dunnett’s *post-hoc* for number of STS, and Tukey’s *post-hoc* for other criteria). * Represents *P* < 0.05, **: *P* < 0.01, ***: *P* < 0.001, ****: *P* < 0.0001. Groups were compared with the control group

To quantitatively investigate cellular reorganization, we measured STS frequency, average STS area, and lumen efficiency from several samples, sections, and images (Supplementary Table S1) using ImageJ software. The TiTOs showed a significant increase in the number of STS compared to the untreated group (Fig. 3A). The most common size range of STSs in the TiTOs was 40–60 μm, while the untreated group had a smaller size range of 20–40 μm (Fig. 3B). This indicates that TGF-β inhibition contributed to the production of more developed tubule-like structures.

We also measured the percentage of area occupied by the tubular structures and found that the TiTOs had a larger STS area compared to the control (Fig. 3C). Furthermore, the number of lumen-bearing STSs were noticeable in TiTOs. In contrast, the control group had either no or very few STSs with lumens (Fig. 3D). We evaluated lumen efficiency by measuring the lumen area relative to the area of the STS with such a lumen. Importantly, TGF-β inhibition resulted in a significant increase in lumen efficiency as shown in Fig. 3E). Overall, these results validated that TGF-β inhibitors enhanced the in vitro reconstruction of tubular structures and significantly improved the yield of TO formation.

### Cellular patterning within testicular organoids

**Fig. 4:**
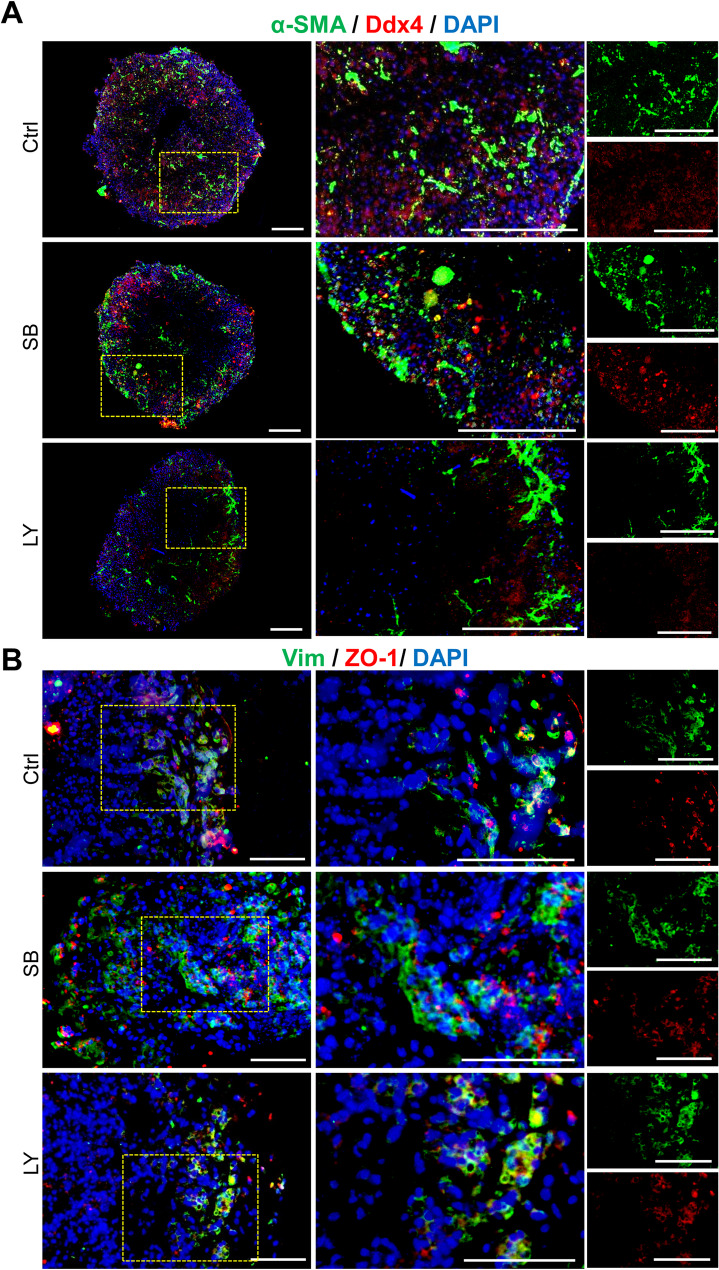
Immunofluorescence analysis of testicular cell-specific markers in TOs. (**A**) Confocal immunofluorescence analysis of α-SMA/Ddx4 expression in whole mounted TiTOs. The results showed the protein expression of spermatogonia and peritubular cell markers (Ddx4 and α-SMA, respectively) in TiTOs. Scale bars: 200 μm. (**B**) Immunofluorescence analysis of Vimentin/ZO-1 expression in TOs. The results indicated protein expression of Sertoli cell and tight junction protein markers (Vimentin and ZO-1, respectively). Cells were counterstained with DAPI to visualize the nuclei. Scale bars: 100 μm

**Fig. 5:**
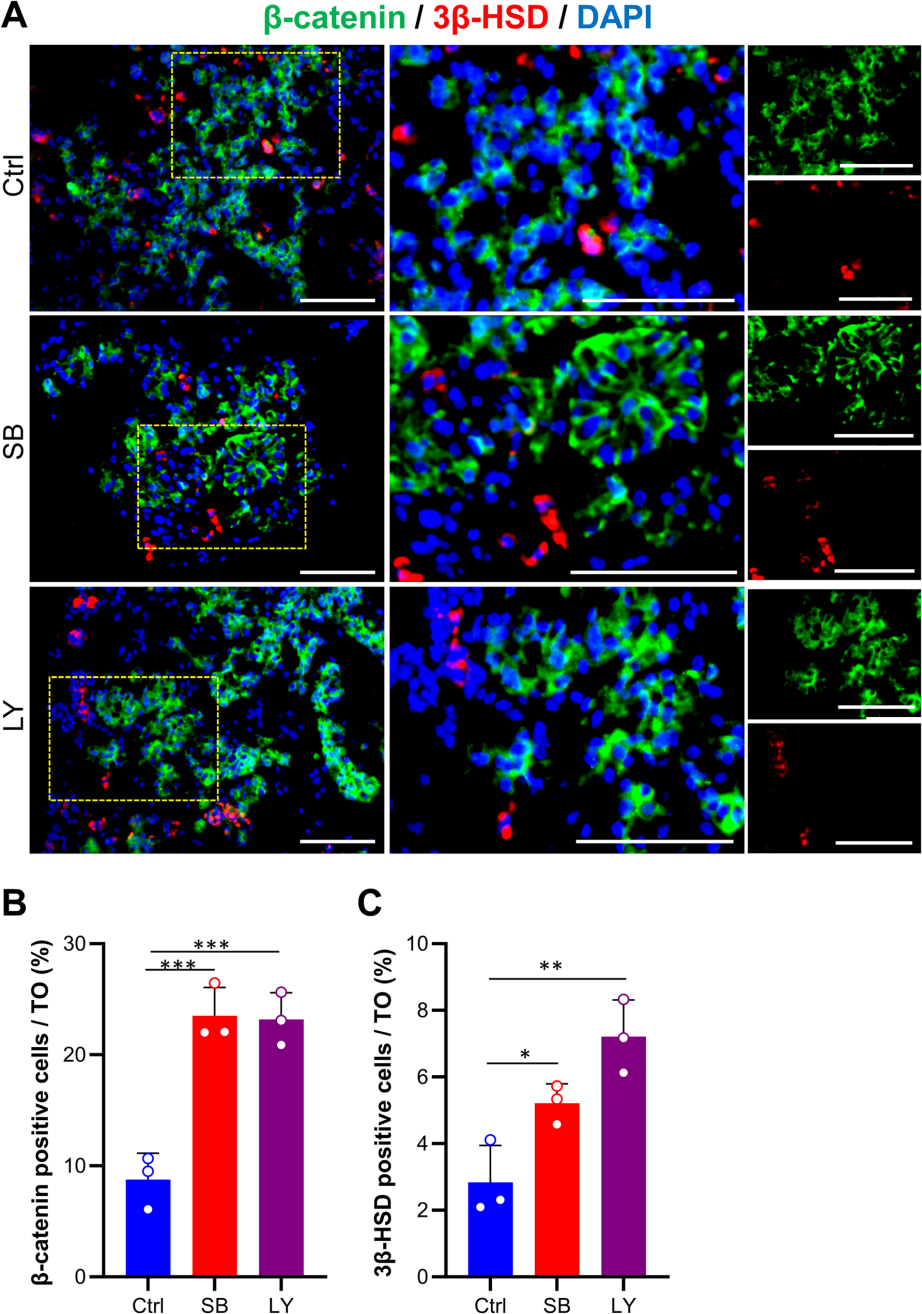
Immunofluorescence analysis of Sertoli and Leydig cell-specific markers in TOs. (**A)** β-catenin was locally expressed within STSs, while 3β-HSD showed interstitial expression in TOs. (**B**) The percentage of β-catenin^+^ cells and β-HSD^+^ cells were significantly higher in the TiTOs. Cells were counterstained DAPI to visualize the nuclei. Data are presented as mean ± SD (*n* = 3) and analyzed by one-way ANOVA and Dunnett’s *post-hoc* test. * Represents *P* < 0.05, **: *P* < 0.01, ***: *P* < 0.001. Scale bars: 100 μm

We performed immunostaining for tissue-specific markers to identify testicular cell types and their spatial organization. As illustrated in the Fig. 4A, α-SMA^+^ peritubular myoid cells were clearly visible in the TiTOs around the STSs. Additionally, we detected Ddx4^+^ spermatogonia cells within tubular compartments in the STSs. In the testis, SCs play a crucial role in the development of the blood-testis barrier (BTB), which is characterized by the expression of ZO-1 indicating the integrity of the tight junction. We examined the coexpression of Vimentin (Vim) and ZO-1 to assess BTB formation. Clusters of Vim^+^ SCs expressing ZO-1 were identified in the TiTOs, suggesting the formation of a BTB-like structure in these organoids (Fig. 4B). The differentiation of SCs and Leydig cells (LCs) is a crucial stage in the formation of seminiferous cords, characterized by the expression of β-catenin and 3β-hydroxysteroid dehydrogenase (3β-HSD) respectively. We discovered 3β-HSD^+^ LCs in the interstitial-like compartments at the periphery of STSs, while β-catenin^+^ cells were in deeper regions within STSs (Fig. 5A). Interestingly, 3β-HSD, despite its abundant presence outside the STS, did not show localization within the STS, as would normally be expected. Quantitative analyses revealed a higher frequency of β-catenin^+^ and 3β-HSD^+^ cells in the TiTOs compared to the control group (Fig. 5B and C). In conclusion, these results clearly demonstrated that both TiTO groups (SB and LY) exhibited structures closely resembling the in vivo testicular tissue architecture while also preserving all major cell types.

**Fig. 6:**
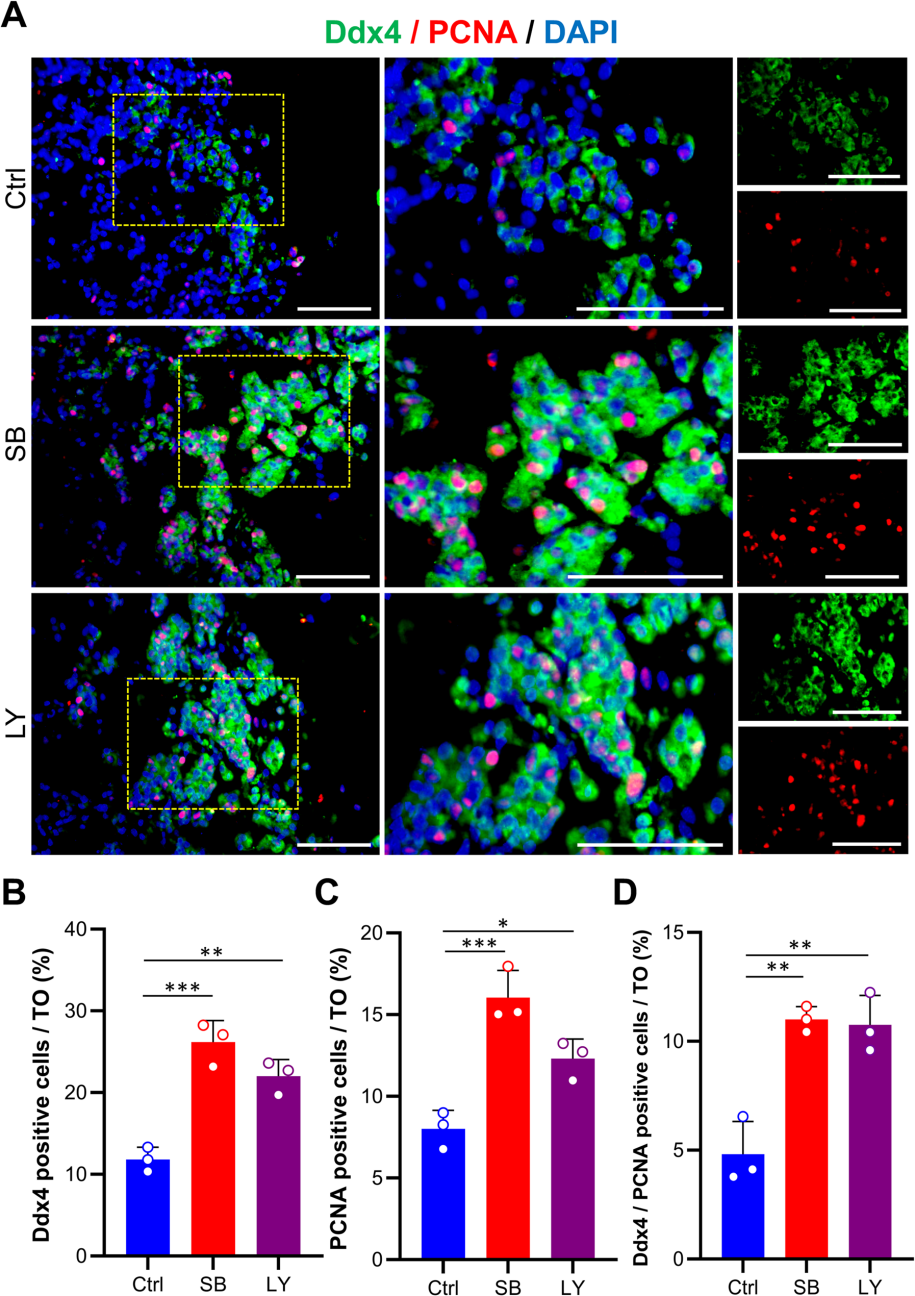
Immunofluorescence analysis of proliferative germ cells in TOs. (**A**) Coexpression of Ddx4/PCNA was strongly detected in the TiTOs, suggesting superior proliferation capacity of spermatogonia in these TOs. (**B**,** C**) Quantified analyses showed a significantly higher number of Ddx4^+^ germ cells and PCNA^+^ proliferative cells in the TiTOs. (**D**) Quantified analyses revealed that TiTOs had a notably higher proportion of proliferative germ cells (Ddx4^+^/PCNA^+^) compared to the control group. The cells were counterstained with DAPI to visualize the nuclei. Data are presented as mean ± SD (*n* = 3) and analyzed by one-way ANOVA and Dunnett’s *post-hoc* test. * Represents *P* < 0.05, **: *P* < 0.01, ***: *P* < 0.001. Scale bars: 100 μm

To assess the proliferation capacity of TiTOs, we evaluated proliferating germ cells by coexpression of Ddx4 and proliferating cell nuclear antigen (PCNA). The results confirmed the expression of these two markers in the TiTOs (Fig. 6A). Quantitative analyses revealed that the TiTOs had a significantly higher number of Ddx4^+^ germ cells (Fig. 6B) and PCNA^+^ proliferating cells (Fig. 6C). Furthermore, the percentage of proliferating germ cells (Ddx4^+^/PCNA^+^ cells) was significantly increased by 2.28-fold and 2.23-fold upon treatment with SB and LY, respectively (Fig. 6D). A negative control for each marker was performed by omitting the primary antibody (Supplementary Fig. 6). These results strongly demonstrated the proliferative effect of TGF-β inhibition on testicular cells within TOs.

### Gonadotropin responsiveness of testicular organoids

**Fig. 7:**
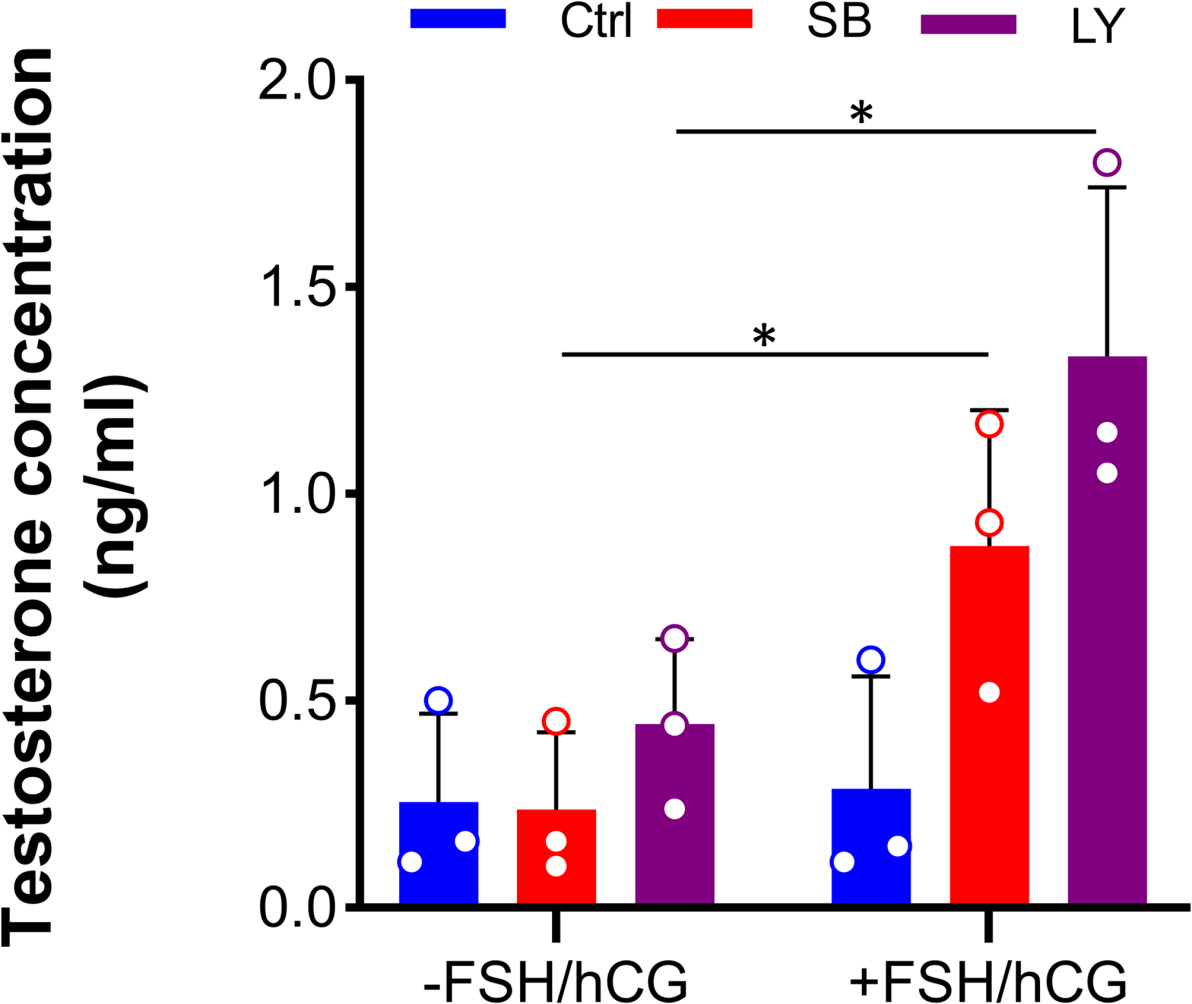
The steroidogenic activity of TOs. Testosterone secretion was significantly higher in the TiTOs compared to the control after treatment with the gonadotropins, indicating improved steroidogenic activity of TiTOs. These data were obtained from three independent experiments, presented as mean *±* SD, and analyzed using *t*-test. * Represents *P* < 0.05

Testosterone synthesis by LCs in response to gonadotropins is a crucial physiological function of the mammalian testes [28, 29]. To explore the steroidogenic activity of TiTOs, testosterone level was measured before and after gonadotropin (FSH/hCG) treatment. The results showed a significant increase in testosterone levels in the TiTOs after FSH/hCG treatment, while no change was observed in the control group (Fig. 7). This indicates that LCs in the TiTOs responded adequately to hormonal stimulation and implies a stimulatory effect of TGF-β inhibition on the physiological function of TOs.

### Intracellular cascades induced by TGF-β inhibition in the testicular organoids

**Fig. 8:**
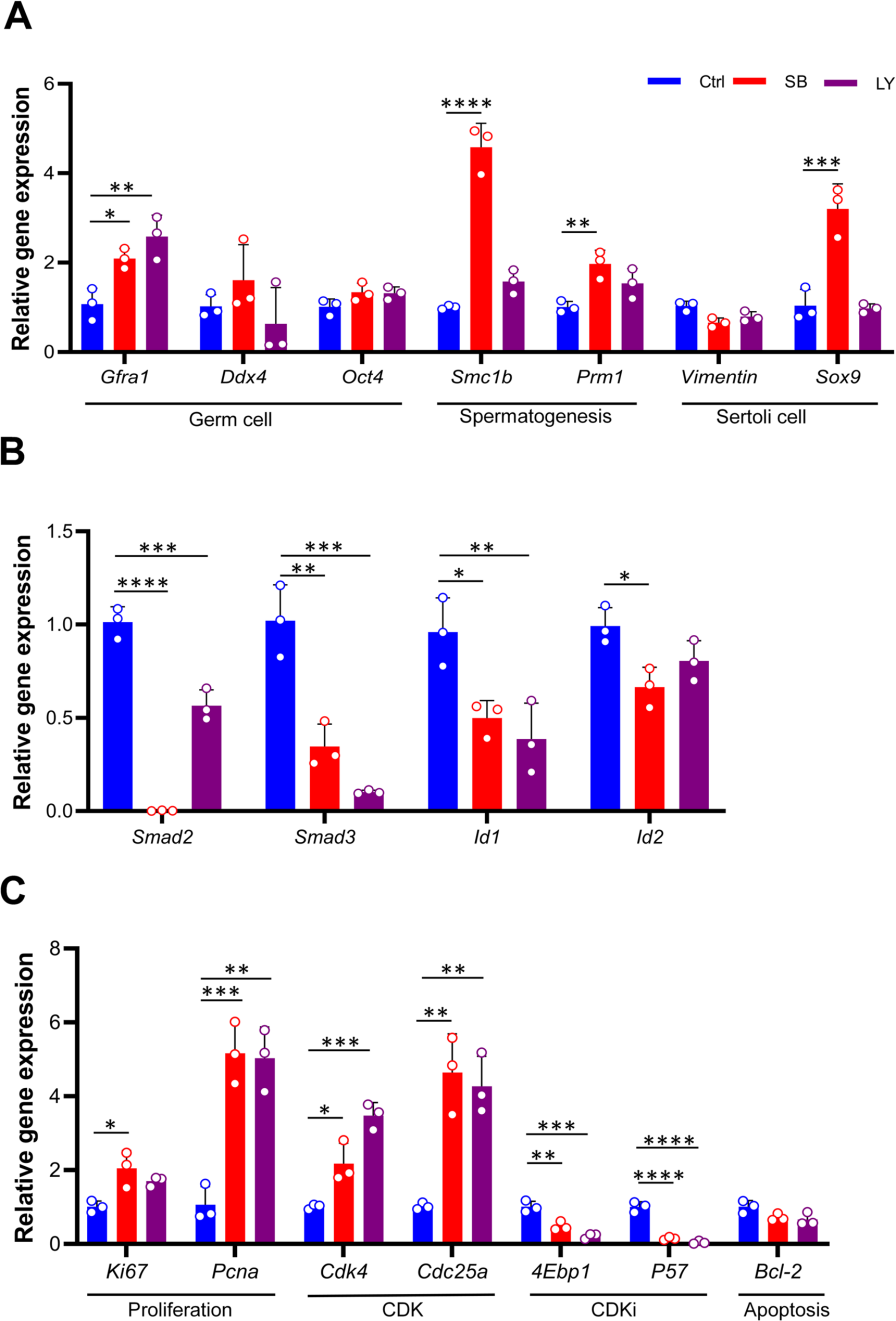
qRT-PCR analyses of key genes specific to germ and Sertoli cells, cell proliferation genes, and TGF-β signaling target genes. qRT-PCR was performed to quantify the expression of genes relevant to germ cells (*Gfrα1*, *Ddx4*, *Oct4*), differentiated germ cells (*Smc1b*, *Prm1*), Sertoli Cells (*Vimentin*, *Sox9*), master regulators of the TGF-β signaling (*Smad2/3*, *Id1/2*), the proliferation genes (*Ki67* and *Pcna*), TGF-β target genes related to proliferation (CDKs, CDKi), and the apoptotic gene (*Bcl-2*). (**A)** Increased levels of *Gfra1*, *Ddx4*, *Smc1b*, *Prm1*, and *Sox9* were observed in the TiTOs compared to the control group. (**B**) The TGF-β signaling target genes *Smad2/3* and *Id1/2* were significantly decreased in TiTOs. (**C**) TiTOs had significantly higher expression of *Pcna* and *Ki67* compared to the control, indicating the stimulatory effect of TGF-β inhibition on proliferation of testicular cells within TOs. Moreover, mRNA expression analyses of the genes associated with the main component of the TGF-β signaling pathway for CDKs and CDKi showed a significant increase in *Cdk4* and *Cdc25a* and a significant decrease in *4Ebp1* and *P57* in the TiTOs compared to the control group. Relative expression levels were normalized against the house-keeping gene G*apdh*. Data are presented as mean ± SD (*n* = 3) and analyzed compared to control group using one-way ANOVA and Dunnett’s *post-hoc* test. ∗*P* < 0.05, ∗∗*P* < 0.01, ∗∗∗*P* < 0.001 and ∗∗∗∗*P* < 0.0001. The fold-change expression in the SB and LY groups was calculated relative to the control group. Each point represents the average value from three independent experiments. Control: untreated TOs

To assess gene expression in TiTOs, we first focused on genes specific to the germ cells and SCs, which are thought to be the organizing hub of the testis. Our qRT-PCR analyses revealed higher levels of *Gfrα1* mRNA, a germ cell-related gene, in TiTOs compared to the control. The expression level of *Ddx4*, another germ cell marker, was also increased in SB-derived TOs. However, no significant difference was observed in the expression levels of *Oct4*, another germ cell-related gene, between TiTOs and control. *Smc1b*, a gene associated with meiotic spermatocytes, exhibited higher expression levels in TiTOs compared to the control. Additionally, increased expression levels of *Prm1*, a post-meiotic marker, were observed in TiTOs, however, the change was not significant in the LY group. Overall, the strong expression of *Smc1b*, together with the increased expression level of *Prm1*, confirmed promotion of spermatogenesis in these organoids. We also observed changes in the expression pattern of the SC-related genes; The mRNA expression level of *Sox9* was increased significantly after treatment with SB but not LY, while no significant change was observed in the expression of *Vimentin* in the TiTOs compared to the control (Fig. 8A).

We next examined the expression of TGF-β receptorI (TGF-βRI) downstream mediators. The expression levels of *Smad2/3*, *Id1*, and, *Id2* were found to be significantly lower in TiTOs, but *Id2* did not change considerably in the LY group (Fig. 8B). To further assess proliferation in TiTOs, we evaluated proliferation-related genes (*Pcna* and *Ki67*). As expected, SB-derived TOs had significantly higher expression of *Pcna* and *Ki67*, compared to control (Fig. 8C). Notably, similar expression patterns were observed in the LY group. We also analyzed the expression of TGF-β target genes involved in the cell cycle process to gain additional insights into the molecular mechanism of TGF-βi-induced cell proliferation in TiTOs (Fig. 8C). Notably, two cell cycle promoting genes (*Cdk4* and *Cdc25a*), known to be the main target of TGF-β signaling, showed significantly higher expression in TiTOs compared to control. Conversely, as expected, these TOs showed a significant decrease in the mRNA expression levels of two cyclin-dependent kinases (CDK) inhibitor (CDKi) genes *4Ebp1* and *P57* as proliferation inhibitor genes. However, gene related to apoptosis, including *Bcl*-2 did not show significant change by TGF-β inhibition. These results highlighted the critical role of TGF-β inhibitors in increasing cell proliferation within TOs by modulating the expression of key cell cycle regulators.

## Discussion

TOs have generated great interest in reproductive biology due to their potential as valuable research models for pharmaco-toxicology and biological studies [1]. However, the development of TOs often suffers from an imbalance between the proliferative spermatogonia and the functional niche cells [9, 21, 30]. Therefore, maintaining a balance of these key cell types is essential for establishing appropriate regional identity in organoids and can be attained through precious regulation of key signaling pathways governing tissue development [30, 31]. Here, we generated TOs with tubule-like structures and interstitial-like compartments by inhibiting the TGF-β signaling pathway and showed that these TOs closely recapitulate in vivo testicular tissue in terms of spatial cell reorganization, expression of specific cell markers, and physiological function.

TGF-β signaling has been shown to regulate testis development, inhibits the proliferation and secretory function of LCs [24, 32–35], and adversely regulates germ cell proliferation in fetal and neonatal mice [36]. The critical role of TGF-β signaling in testicular development makes it a potential contributor to testicular diseases and infertility [37]. Moreover, patients with SC-only syndrome and LC hyperplasia exhibit elevated mRNA and protein expression of TGF-β1 signaling mediators, particularly Smad2/3. These findings strongly imply the involvement of TGF-β in infertility, such as LC hyperplasia [37, 38]. Accordingly, several pharmacological interventions, such as antibodies and small molecule inhibitors, targeting different components of the TGF-β signaling pathway, have shown promising results in both preclinical and clinical studies [39–43]. In line with these studies, we have previously demonstrated that inhibition of TGF-β signaling promotes spermatogenesis by expanding undifferentiated spermatogonia while preserving their differentiation potential. In particular, the TGF-β inhibitor SB has been shown to increase spermatogonia proliferation in both in vitro and in vivo [44]. These findings led us to hypothesize that the proliferative effect of TGF-β inhibition on the key cellular components involved in organoid development, would enhance the capacity and efficiency of TO development. To address our hypothesis, we examined the effects of two TGF-β inhibitors on cellular reorganization and TO formation using a well-known Matrigel-based culture system [6, 45]. In our study, after culture of testicular cells for 5 days, the culture medium was supplemented with SB or LY, small molecule inhibitors of TGF-βRI kinase [41, 46] during an additional 11-days culture for TO development. The main feature of TiTOs was their compartmentalized structures resembling the seminiferous cords and interstitial space in testis tissue, as evidenced by the formation of tubular structures with distinct expression patterns of tissue-specific markers.

In male gonad development, aggregation of SCs-germ cells and the enlargement of these structures are important initial events in the formation of seminiferous cord structures [9, 21, 47]. Accordingly, many research groups have attempted to produce TOs through the formation of multicellular aggregates. However, these structures were far enough away from the complex architecture of the testis with its integrated discrete compartments [1, 4, 10]. According to our histological evaluations, the TiTOs exhibited a well-organized architecture featuring tubules with lumen-like structures closely resembling the compartmentalized architecture characteristic of in vivo testicular tissue. Interestingly, some structures in the SB and LY groups revealed in-vivo-like patterning, as the primary sex cords appeared during testis development, suggesting the possibility of initiating the formation of tubular structures. Notably, TGF-β inhibitors enhanced the cellular reorganization within TOs as evidenced by the increased number, size and area of STSs with a distinct expression pattern of the tissue-specific markers. Only a few studies have successfully met al.l the criteria for generation of testicular organoids with compartmentalized and complete testis tubular/interstitial structures, including at least three major cell types of the testis [48–50]. More importantly, a noticeable portion of the tubules in TiTOs were distinguishable with lumen-like inner spaces, a finding that has been rarely achieved in previous studies [49]. Research has frequently highlighted the importance of a central lumen in replicating the structure and functionality of the native organs [51]. Overall, the enlargement of STSs, along with the formation of lumen-like structures in our study, may serve as indicators of post-pubertal maturity [49].

The tubule-like structures in the SB and LY groups displayed similar protein expression patterns and spatial arrangements of tissue-specific markers. We found β-catenin^+^ SCs within the STSs and 3β-HSD^+^ LCs between the STSs in distinct interstitial-like compartments primarily located in the peripheral area of the STSs, indicating compartmentalization of tubular and interstitial cell types in the TiTOs. These results are consistent with previously described expression patterns for these proteins in TOs [8, 9, 14, 19, 49].

Our study showed increased proliferation of testicular cells through the inhibition of the TGF-β pathway that may be the main cause of the enhanced development of TOs as evidenced by the increased expression of genes associated with cell proliferation, such as *Pcna* and *Ki67* in TiTOs. The PCNA marker was also more abundantly expressed in these TOs at protein level, indicating the stimulatory effect of TGF-β inhibition on testicular cell proliferation. The increased number of Ddx4^+^/PCNA^+^ cells further indicate the promotion of proliferative spermatogonia through TGF-β inhibition. Furthermore, a significant increase in the expression of *Gfrα1* implies a larger pool of spermatogonia cells in TiTOs [52]. These results were consistent with the previous study that have shown the TGF-β as a negative regulator of germ cell proliferation in mice [36]. In addition, our study showed that inhibition of TGF-β increased the number of 3β-HSD^+^ LCs compared to the untreated control, as expected with its known inhibitory effect on LC proliferation and differentiation [24, 32, 35, 53]. Similarly, Zhang et al., have reported that supplementing the medium of cultured testicular cells within a collagen matrix with KSR resulted in increased proliferation and differentiation of SCs and spermatogonia cells, ultimately leading to the formation of TOs with tubule-like structures (9). Other studies have also documented that generation of TOs with tubule-like structures can be improved by stimulating cell proliferation using exogenous factors [54–57]. These observations collectively suggested that inhibiting TGF-β pathway preserves germ cells and enhances spermatogonia proliferation in TOs.

We found that SB and LY effectively blocked the TGF-β/Smad2/3 pathway by reducing *Smad2/3* and *Id1/Id2* mRNA levels which are direct targets of this signaling pathway. In the TGF-β signaling pathway, Smad2 and Smad3 are important downstream mediators of the TGF-βR1 that regulate the expression of the target genes CDK and CDKi [58]. This signaling pathway plays a crucial role in testicular development and spermatogenesis through the regulation of these target genes [34]. We found that TGF-β inhibitors promoted cell proliferation by down-regulating CDKi and up-regulating CDKs. These results were consistent with our previous work showing that SB stimulated cell proliferation through the regulation of CDKs and CDKi [44]. Moreover, other research have demonstrated the inhibitory effect of TGF signaling on cell proliferation through upregulation of the CDK inhibitors *P15* and *P21* [56] and downregulation of the *Cdk4* [59] and *Cdc25a* [60].

The TiTOs were gonadotropin responsive, as previously reported in some studies to indicate the functionality of TOs [3, 4, 7, 18, 49, 61]. However, the steroidogenic activity of TOs has not been shown in many previous studies, even those with well-organized TOs [2, 5, 6, 8, 50, 62]. Our finding validated the previously reported inhibitory effect of TGF-β1 on testosterone secretion [33, 34, 63] and agreed with the work of Yang et al., which demonstrated that the inhibitory effect of TGF-β on the steroidogenic activity of LCs could be reversed by a TGF-βR1 inhibitor [33]. Several lines of evidence indicate that TGF-β1 regulates LC function by suppressing proliferation and hindering testosterone production through inhibition of key steroidogenic genes such as *3β-HSD* [24, 32, 34, 64]. Accordingly, the higher number of 3β-HSD^+^ LCs, a marker of the steroidogenic pathway, may be responsible for the increased testosterone production observed in TiTOs in our work.

Testosterone is crucial for the initiation and maintenance of spermatogenesis. Hence, the ability to secrete testosterone is a well-known indicator of spermatogenesis [28, 29]. Consequently, failure to accurately replicate a physiologically relevant testicular surrogate has been reported as a possible cause of incomplete spermatogenesis in TOs [49]. This underscores the significant connection between testosterone secretion and spermatogenesis. Remarkably, significant increases in the expression of *Smc1b* and *Prm1* in our TOs confirmed the promotion of in vitro spermatogenesis, a finding that has not been reported in most of previous studies [6, 15, 18, 49]. Totally, the capability of TiTOs to produce testosterone and express meiosis-related genes, provides compelling evidence to support in vitro spermatogenesis within these organoids and underscores the potential impact of TGF-β inhibition on germ cell differentiation. Nevertheless, more investigation is needed to properly ascertain the efficiency of in vitro spermatogenesis in TiTOs. These observations reinforce our finding that TGF-β inhibition can promote testicular cells reorganization and TO development by stimulating cell proliferation and supporting spermatogenesis within TOs.

This surpasses previous efforts for TO development from testicular cells cultured in the Matrigel-based core-shell system, which revealed no lumen-like structures and no steroidogenic activity in their TOs [6, 8, 62]. Moreover, compared to recent efforts that employed complicated advanced technologies to guide cell organization [3, 49], TGF-β inhibition may provide a versatile and cost-effective strategy to produce TOs across different culture systems. The well-organized TOs obtained through TGF-β inhibition could offer a valuable tissue model for non-invasive research on testicular endocrinology. The small molecule inhibitors studied in our research are currently being assessed for other indications in various preclinical and clinical trials [40, 65, 66]. Hence, these small molecules may offer promising therapeutic targets for addressing male infertility issues in the future. Given our reliance on these two common TGF-β inhibitors, future studies should investigate additional, more specific small molecules to better understand the pathway and improve therapeutic applications in testicular tissue engineering. Although our study utilized immature testicular cells from animal sources due to their high regenerative capacity, future research should focus on validating this model with ethically sourced prepubertal human testicular tissue to enhance translational relevance.

## Conclusion

This study presents an efficient method for reconstructing tubule-like structures from primary testicular cells and developing functional TOs by targeting the TGF-β signaling pathway. By using small molecule TGF-β inhibitors (SB or LY) we have successfully developed TOs with compartmentalized architecture and steroidogenic activity, which supported the survival and meiotic promotion of early germ cells. This study provided a deeper understanding of the detailed role of TGF-β inhibition in testis reconstruction and introduce a versatile tool that may advance new approaches for generation of TO models.

## Electronic supplementary material

Below is the link to the electronic supplementary material.

Supplementary Material 1

## Abbreviations

TOs: Testicular Organoids
TGF-β: Transforming growth factor-beta
SB: SB431542
LY: LY2157299
STSs: Spherical-tubular structures
TiTOs: TGF-β inhibitor-derived testicular organoid
SCs: Sertoli Cells
LCs: Leydig Cells
3β-HSD: 3β-hydroxysteroid dehydrogenase
CDK: Cyclin-dependent kinases
