# Supplementary material for: Harnessing TGF-β signaling to improve testicular organoid development from dissociated testicular cells
Source: Stem Cell Res Ther. 2025 Aug 20;16:443. doi: 10.1186/s13287-025-04513-0 (PMC12366404; doi:10.1186/s13287-025-04513-0)
Supplement: Supplementary file 1 — Supplementary Material 1 [file 13287_2025_4513_MOESM1_ESM.docx]

**Supplementary information**


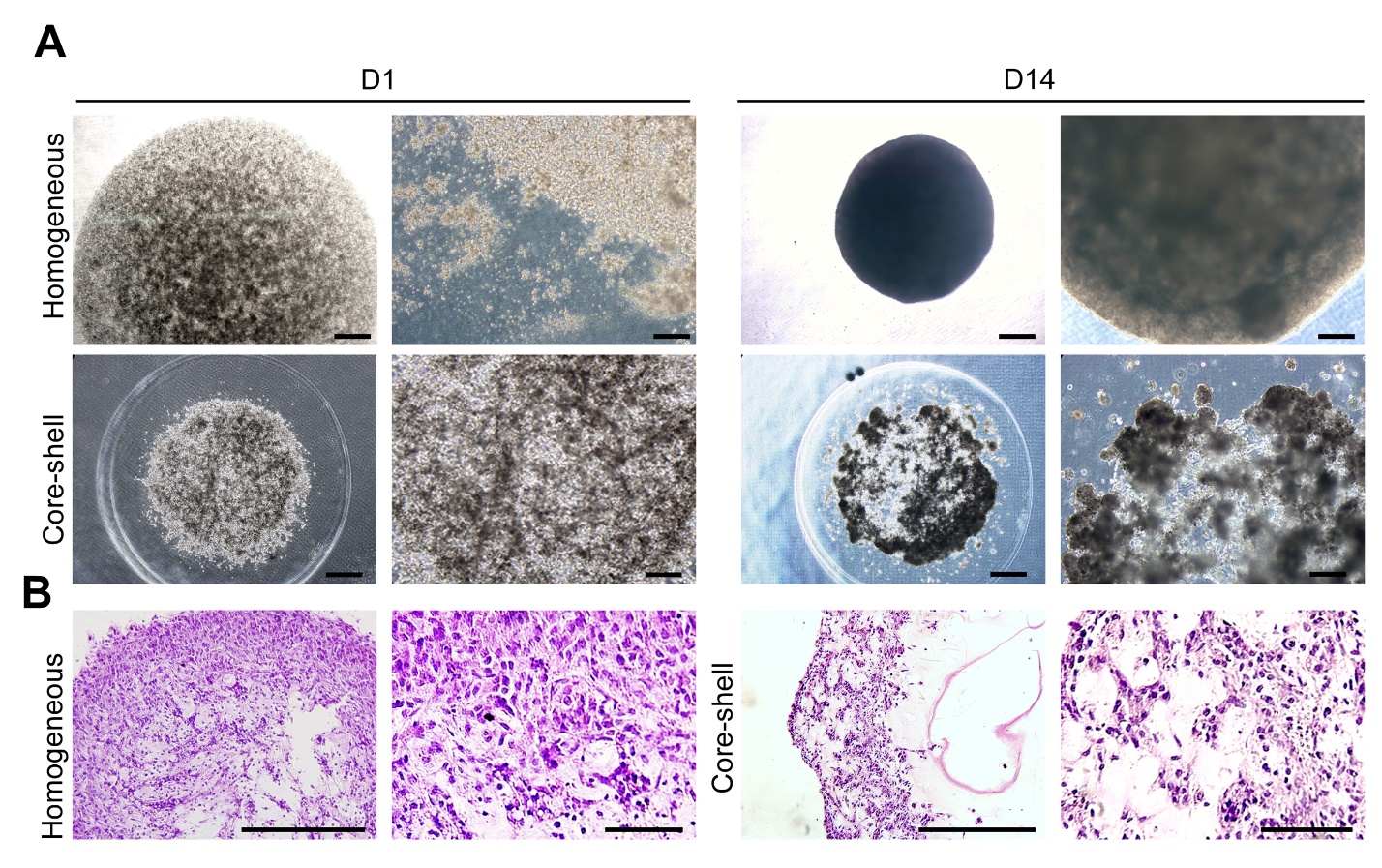


Supplementary Fig. 1 Establishment of TO culture system. Representative brightfield images of mouse testicular cells after 3D culture in Matrigel with two configurations, core-shell hydrogel and homogeneous hydrogel for 1 and 14 days. (**A)** Testicular cells self-assembled after 14 days in core-shell, whereas cultured cells in homogeneous hydrogel formed a tightly packed aggregate. Scale bars: 500 µm, high magnification: 200 µm. (**B)** In contrast to the core-shell group, no cellular reorganization was detected in the histological sections of homogeneous hydrogel after 14 days of culture. Right panel: High magnification. Scale bars: 200 µm, high magnification: 50 µm.


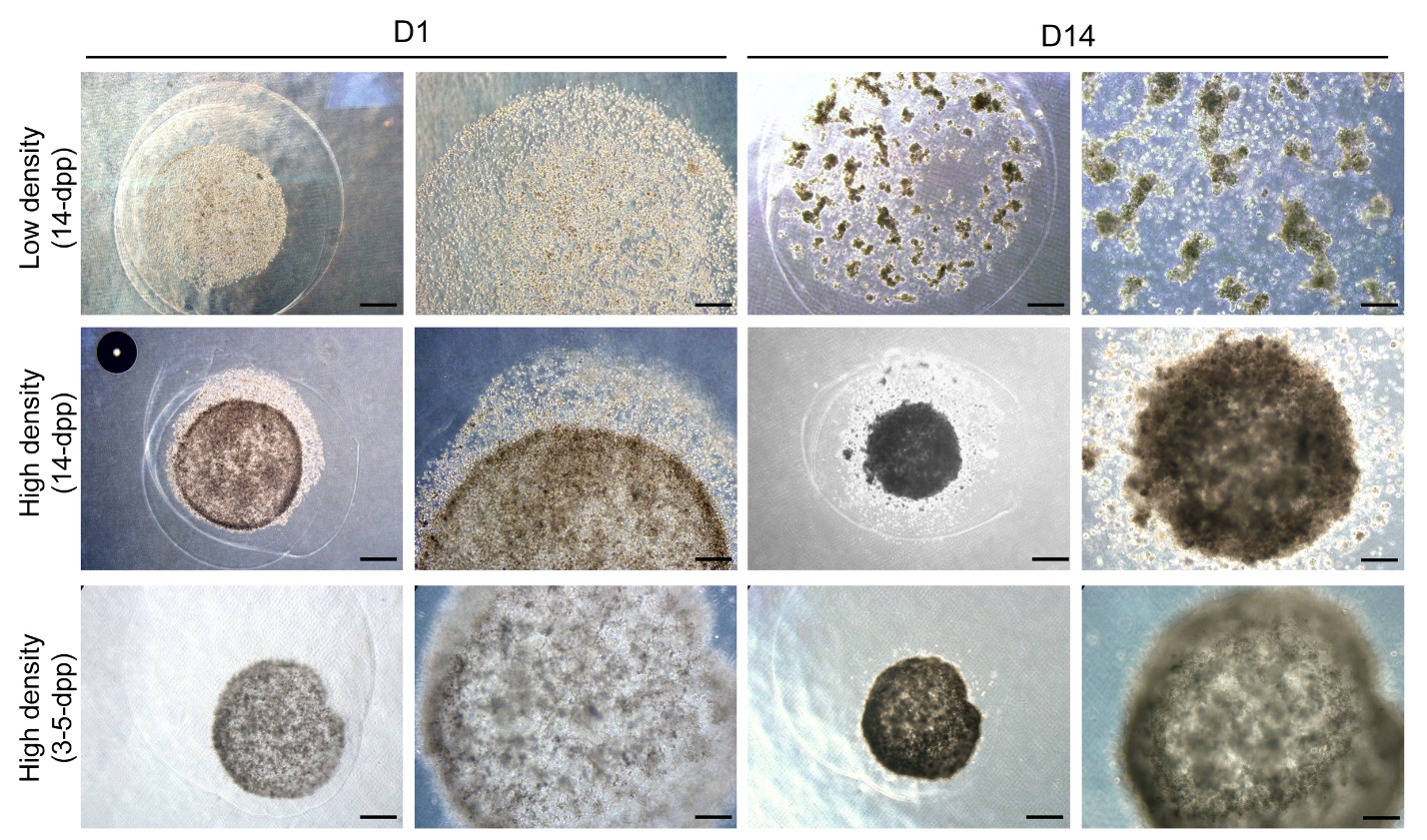


Supplementary Fig. 2 Determination of proper cell origin and density for TO culture. Phase contrast images of the testicular cells isolated from mice with different ages (neonatal and prepubertal cells from mice aged 3-5 and 14 days) and cultured with low and high-density (22 and 44 million cells/ml) for 1 and 14 days in core-shell system. In contrast to the discrete aggregates derived from the low-density culture, the high-density culture revealed a better cellular organization into a complex of integrated compartments. Moreover, the cells isolated from 14-dpp mice had a greater ability for self-assembly. Right panel: High magnification. dpp: days postpartum. Scale bars: 500 µm, high magnification: 200 µm.


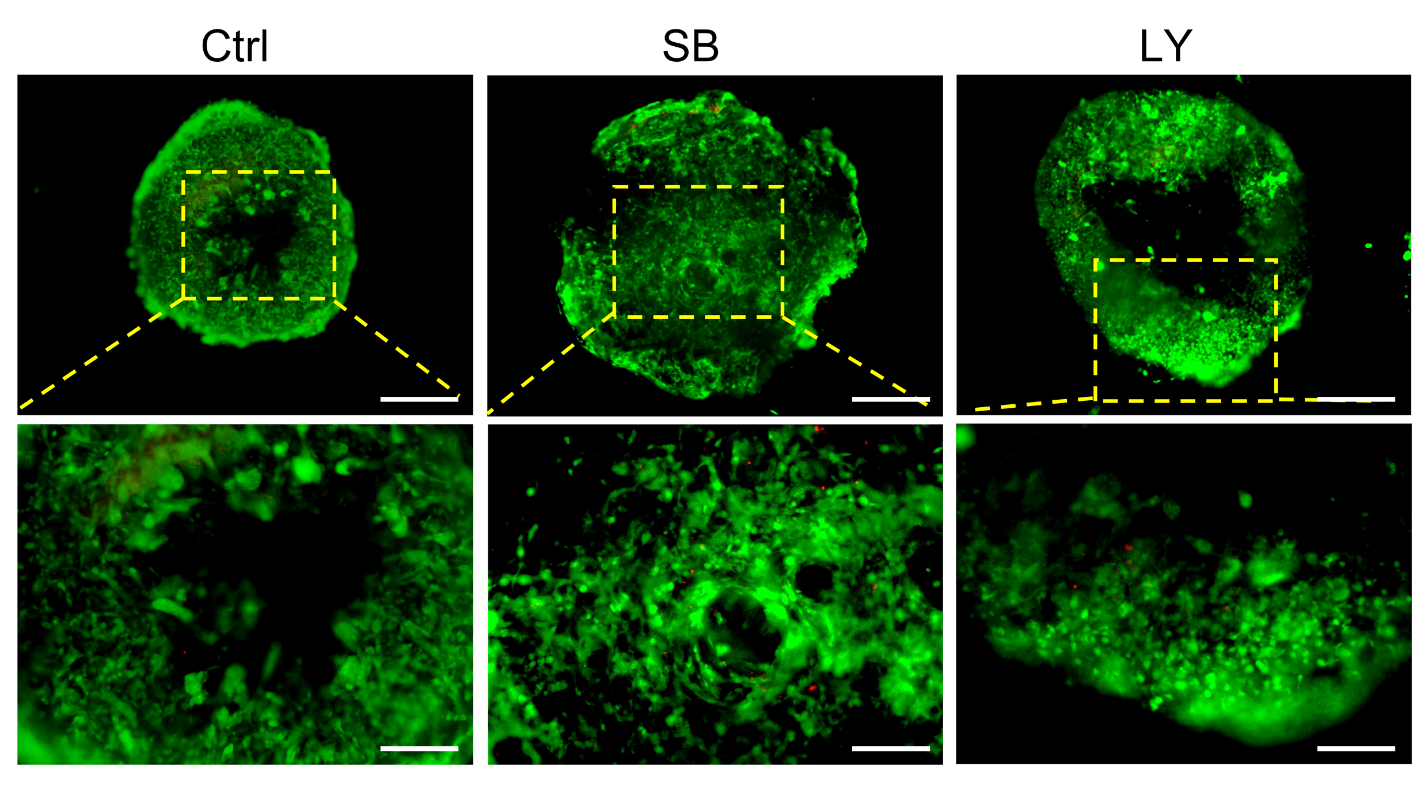


Supplementary Fig. 3 Viability assessment of testicular cells in TOs after treatment with TGF-β inhibitors. Live/Dead staining of the TOs revealed no significant cell death in all three groups over the entire 16-day culture. Scale bars: 500 µm, high magnification: 200 µm.


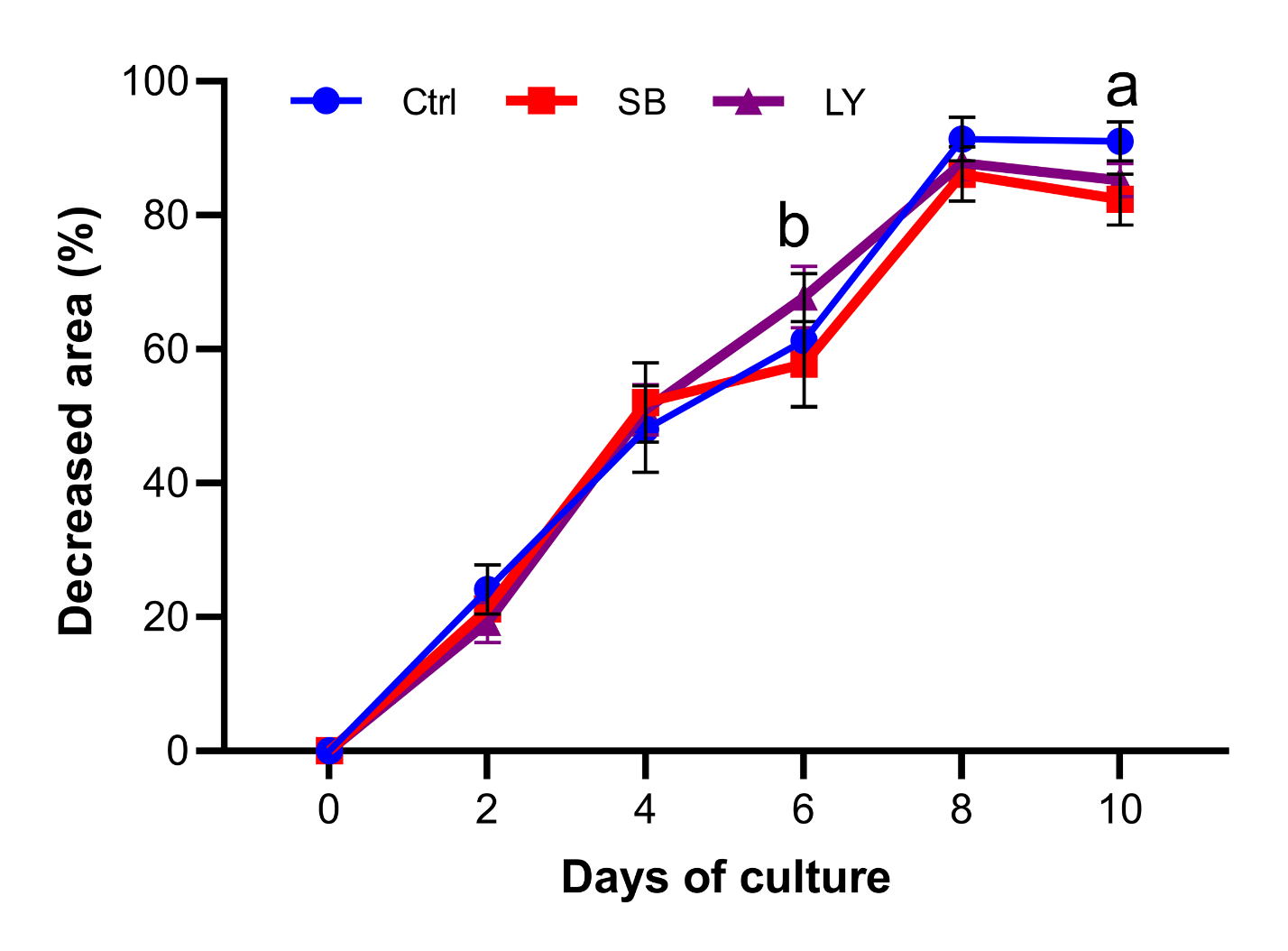


Supplementary Fig. 4 Migration profile of testicular cells during the 10-day culture for TO development. The area occupied by cells relative to the total area of TOs was measured at different days. All groups showed a similar cell migration pattern; however, some differences were observed on days 6 and 10. Data are presented as mean±SD (n=3), and analyzed by two-way ANOVA and Tukey’s *post-hoc* test. a, represents significancy of ctrl vs. SB, P < 0.05 and b, represents SB vs. LY, P < 0.05.


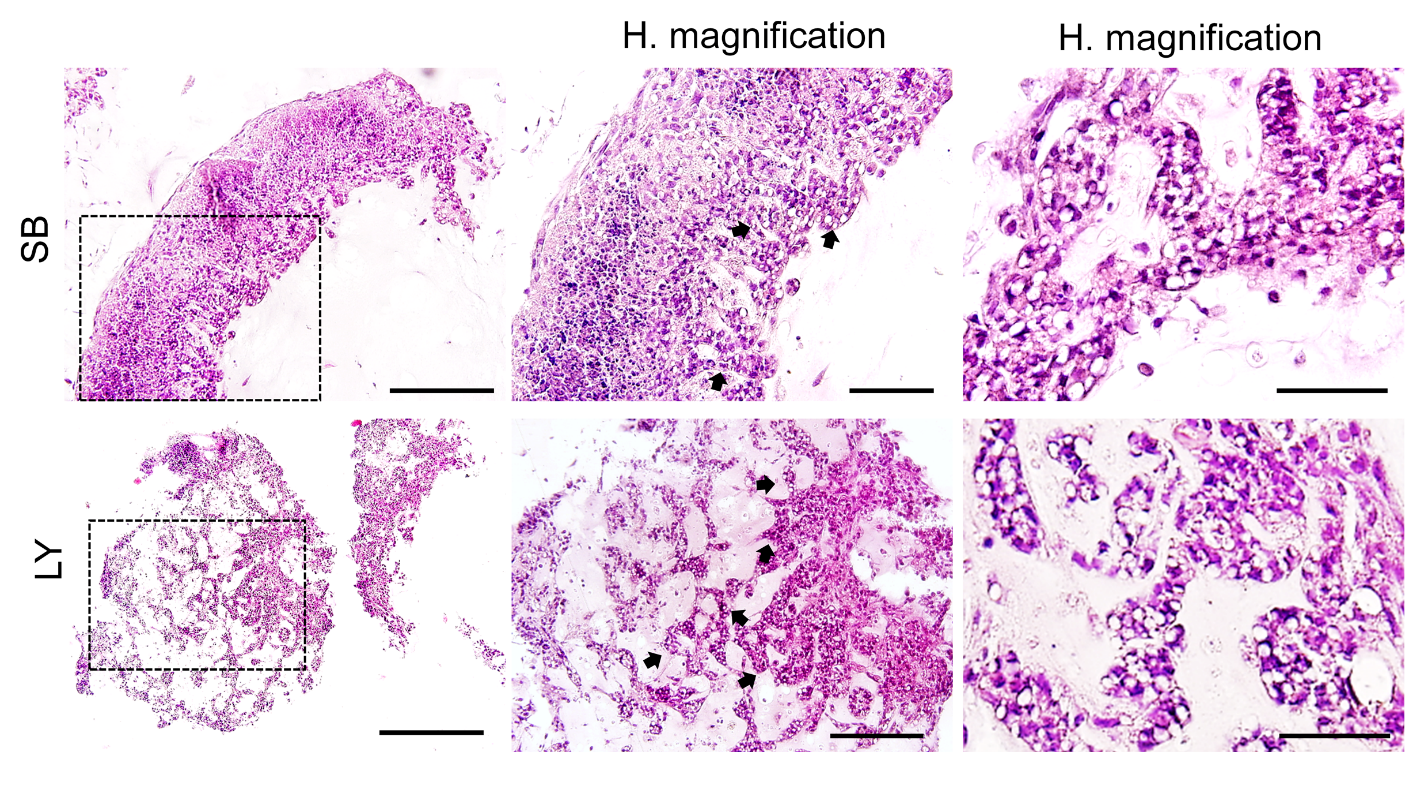


Supplementary Fig. 5 Reconstruction of cord-like structures in TOs after treatment with TGF-β inhibitors. Cord-like structures resembling the primary sex cords in testis development were detected in the histological sections of both SB and LY treated TOs as indicated by the black arrows. Scale bars: 500 µm, high magnification: 200 µm and 50 µm.


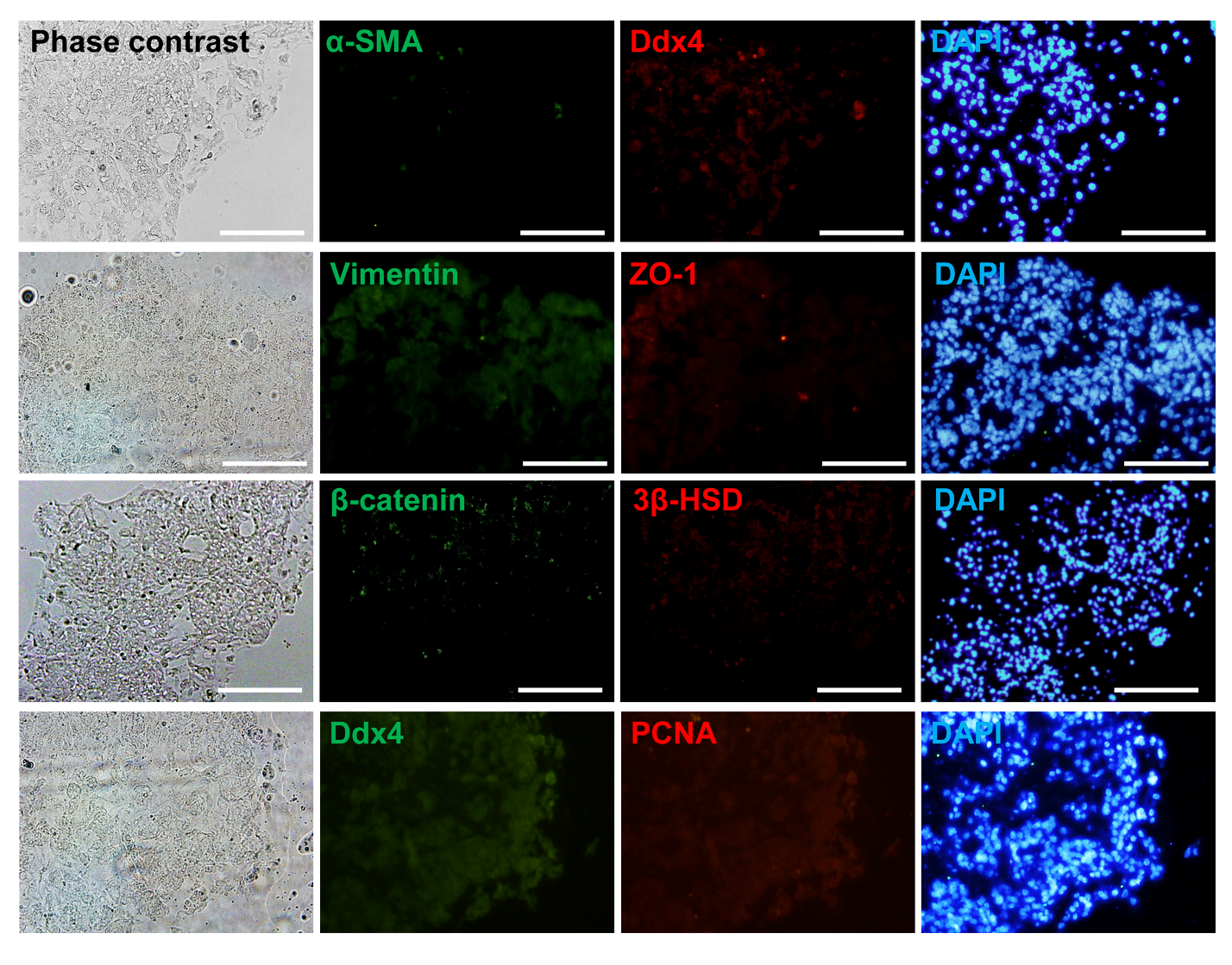


Supplementary Fig. 6 Negative immunostaining of TOs in the absence of the primary antibodies. Negative controls stained in the absence of the primary antibodies were included in each set of immunostaining experiments. Scale bars: 200 µm.

**Supplementary Table 1: Information on sample size for quantified analyses of histological evaluations.**

| Group | No. Biological replicates | No. Organoids | No. Sections | No. Fields | No. Total content STSs |
| --- | --- | --- | --- | --- | --- |
| Ctrl | 3 | 6-8 | 10 | 36 | 42 |
| SB | 3 | 6-8 | 13 | 49 | 184 |
| LY | 3 | 6-8 | 10 | 38 | 200 |

**Supplementary Table 2:** **Information on sample size for quantified analyses of β-catenin/3β-HSD immunostaining.**

| Group | No. Biological replicates | No. Organoids | No. Sections | No. Fields | No. DAPI^+^ | No. β-catenin^+^ | No. 3β-HSD^+^ |
| --- | --- | --- | --- | --- | --- | --- | --- |
| Ctrl | 3 | 3 | 6 | 24 | 3790 | 303 | 98 |
| SB | 3 | 3 | 6 | 26 | 5406 | 1252 | 270 |
| LY | 3 | 3 | 6 | 20 | 2964 | 699 | 219 |

| Group | No. Biological replicates | No. Organoids | | No. Sections | No. Fields | No. DAPI^+^ | No. Ddx4^+^ | No. PCNA^+^ | No. Ddx4^+^/PCNA^+^ |
| --- | --- | --- | --- | --- | --- | --- | --- | --- | --- |
| Ctrl | 3 | | 3 | 6 | 18 | 3677 | 423 | 306 | 155 |
| SB | 3 | | 3 | 6 | 19 | 5881 | 1545 | 939 | 647 |
| LY | 3 | | 3 | 6 | 19 | 6040 | 1378 | 763 | 650 |

**Supplementary Table 3**: **Information on sample size for quantified analyses of Ddx4/ PCNA immunostaining.**

**Supplementary Table 4: List of primers used for gene expression analyses.**

| **Gene name** | **Forward** | **Reverse** |
| --- | --- | --- |
| ***Smad 2*** | **F: 5' TGTCGTCCATCTTGCCATTCA 3'** | **R: 5' TTCTGCTCTCCACCACCTGC 3'** |
| ***Smad3*** | **F: 5' TGCGAGAAGGCG GTCAAGAG 3'** | **R: 5' ACTTGGTGTTCACGTTCTGCG 3'** |
| ***Id1*** | **F: 5' GGTCCGAGGCAGAGTATTACA 3'** | **R: 5' CCTGAAAAGTAAGGAAGGGGGA 3'** |
| ***Id2*** | **F: 5' GTCTGAAGTCGGGACCAC 3'** | **R: 5' GTCTGAAGTCGGGACCAC 3'** |
| ***Gfrα1*** | **F: 5' AATTGTCTGCGTATCTACTGG 3'** | **R: 5' ACATCTGATATGAACGGGAC 3'** |
| ***Ddx4*** | **F: 5' TATGTGCCTCCCAGCTTCAGTA 3'** | **R: 5' CTGGATTGGGAGCTTGTGAAGA 3'** |
| ***Oct4*** | **F: 5' GATGCTGTGAGCCAAGGCAAG 3'** | **R: 5' GGCTCCTGATCAACAGCATCAC 3'** |
| ***Smc1b*** | **F: 5' ACACAGTTTTCGGCCTGCTC 3'** | **R: 5' CCAGAGTCAACACATGGCTG 3'** |
| ***Prm1*** | **F: 5' GATGCACAGAATAGCAAGTCC 3'** | **R: 5' GTGGCATTGTTCCTTAGCAG 3'** |
| ***Vimentin*** | **F: 5' TACAAGTCCAAGTTTGCTGACC 3'** | **R: 5' TTTAAGGGCATCCACTTCACAG 3'** |
| ***Sox9*** | **F: 5' AGGAAGCTGGCAGACCAGTA 3'** | **R: 5' TCCACGAAGGGTCTCTTCTC 3'** |
| ***Ki67*** | **F: 5' AGAGCCTTAGCAATAGCAACG 3'** | **R: 5' GTCTCCCGCGATTCCTCTG 3'** |
| ***Pcna*** | **F: 5' TTTGAGGCACGCCTGATCC 3'** | **R: 5' GGAGACGTGAGACGAGTCCAT 3'** |
| ***Cdk4*** | **F: 5' ATGGCTGCCACTCGATATGAA 3'** | **R: 5' TCCTCCATTAGGAACTCTCACAC 3'** |
| ***Cdc25a*** | **F: 5' ACAGCAGTCTACAGAGAATGGG 3'** | **R: 5' GATGAGGTGAAAGGTGTCTTGG 3'** |
| ***4Ebp1*** | **F: 5' GGGGACTACAGCACCACTC 3'** | **R: 5' CTCATCGCTGGTAGGGCTA 3'** |
| ***P57*** | **F: 5' CGAGGAGCAGGACGAGAATC 3'** | **R: 5' GAAGAAGTCGTTCGCATTGGC 3'** |
| ***Bcl-2*** | **F: 5' GTCGCTACCGTCGTGACTTC 3'** | **R: 5' CAGACATGCACCTACCCAGC 3'** |
|  |  |  |
